# Two-Step Estimation Procedure for Parametric Copula-Based Regression Models for Semi-Competing Risks Data

**DOI:** 10.3390/e27050521

**Published:** 2025-05-13

**Authors:** Qingmin Zhang, Bowen Duan, Małgorzata Wojtyś, Yinghui Wei

**Affiliations:** 1 Yunnan Key Laboratory of Statistical Modeling and Data Analysis, Yunnan University, Kunming 650500, China; xylyzhang@163.com (Q.Z.); bwduan1202@gmail.com (B.D.); 2 Centre for Mathematical Sciences, School of Engineering, Computing and Mathematics, University of Plymouth, Plymouth PL4 8AA, UK; malgorzata.wojtys@gmail.com

**Keywords:** semi-competing risks, copula, right censoring, Amsterdam Cohort Study, BOBYQA

## Abstract

Non-terminal and terminal events in semi-competing risks data are typically associated and may be influenced by covariates. We employed regression modeling for semi-competing risks data under a copula-based framework to evaluate the effects of covariates on the two events and the association between them. Due to the complexity of the copula structure, we propose a new method that integrates a novel two-step algorithm with the Bound Optimization by Quadratic Approximation (BOBYQA) method. This approach effectively mitigates the influence of initial values and demonstrates greater robustness. The simulations validate the performance of the proposed method. We further applied our proposed method to the Amsterdam Cohort Study (ACS) real data, where some improvements could be found.

## 1. Introduction

In follow-up biomedical studies, objects may experience two types of events: a non-terminal event (e.g., disease relapse) or a terminal event (death). The occurrence of a non-terminal event can preclude the occurrence of a terminal event, but not vice versa. This is referred to as semi-competing risks (Fine et al. [1]).

There are two conventional statistical methods for analyzing semi-competing risks data: the random effects model and the copula-based model. As in the study by Xu et al. [2], the random effects model usually adopts a shared frailty non-negative random variable to generate the joint distribution. In contrast, Shih et al. [3] showed that the copula-based model constructs the joint distribution by connecting two marginal distributions, and the copula parameters can explicitly estimate the dependence strength. For the copula-based model, the marginal distribution of times to events conditioned on covariates can be specified independently, and the covariate effects on marginal events can be accessed directly. Moreover, Ramadan et al. [4] highlighted that the copula-based model can capture asymmetric and nonlinear connections between variables and simplify the computation of certain probability measures. Using the copula function, Fayomi et al. [5] proposed the BFGMLG family to model bivariate continuous data with heavy-tailed and skewed distributions; they applied this to environmental, medical, and computer science data. Haj Ahmad et al. [6] proposed a bivariate modified extended exponential (BMExE) distribution and adopted this model to study the relationship between processor and memory reliability in data science. Ramadan et al. [4] introduced the BAPB-XII distribution and applied it to engineering data. In this article, we consider the copula model for semi-competing risks data.

Fine et al. [1] proposed estimators of the association parameter from the concordance-estimating function and used a novel plug-in estimator for the unspecified marginal distribution of a non-terminal event. Jiang et al. [7] proposed another copula-based estimator with better properties based on the principle of pseudo-self-consistency. Peng and Fine [8] considered a class of time-varying coefficient semiparametric regression models for marginals and copulas, and they proposed an estimator based on the moment method. Hsieh and Huang [9] analyzed semi-competing risks data based on the conditional likelihood approach. They adopted semiparametric transformation models as the marginal regressions, which include the popular proportional hazards and proportional odds models as special cases.

For the copula-based model, Shih and Louis [3] proposed a two-stage procedure for the estimation of parameters in the marginal distribution and copula function. In the first stage, the parameters for the marginal distributions are estimated independently for each event, disregarding the dependencies between events; in the second stage, the parameter associated with the copula is estimated via maximizing the log-likelihood using the plug-in estimation of the marginals. However, this approach may not be suitable for the copula-based models for semi-competing risks data, as a terminal event may dependently censor the non-terminal event. Chen [10] considered a nonparametric maximum likelihood estimation (MLE) approach, which involves simultaneously estimating the parameters that are associated with the marginals and the copula via maximization of the log-likelihood function. Arachchige et al. [11] provided a two-stage pseudo-maximum likelihood estimation approach (PMLE). More specifically, in the first stage, they obtained a consistent estimate of the marginal of the terminal event; in the second stage, they estimated the marginal of the non-terminal event and the copula parameters simultaneously by maximizing the pseudo-likelihood function with the plug-in estimation of the terminal event. Sun et al. [12] proposed a two-step method: in the first step, they obtained the parameter estimation separately as the initial value while, in the second step, they obtained all parameters of the marginal distribution and copula function simultaneously through a joint distribution. They called this procedure a two-step procedure. This method can mitigate the influence of initial values, and is adopted in our article.

In addition, due to the complex structure of a copula-based objective function, the derivative information is unavailable, unreliable, or computationally expensive to obtain. In such cases, derivative-free optimization (DFO) methods serve as a viable alternative. This article adopts the Bound Optimization by Quadratic Approximation (BOBYQA) algorithm proposed by Powell [13]. As summarized by Ragonneau [14], Powell pioneered his first DFO method using conjugate directions in 1964. From the 1990s onward, he developed five model-based DFO algorithms, each tailored to distinct problem classes: COBYLA for nonlinearly constrained problems, UOBYQA and NEWUOA for unconstrained problems, BOBYQA for bound-constrained problems, and LINCOA for linearly constrained problems. However, a key limitation of BOBYQA is its limited scalability to high-dimensional problems.

In this article, we consider the parametric copula-based regression model for semi-competing risks data. Fully parametric approaches are appealing because they are generally identifiable, can produce smooth survival functions, and give a direct interpretation [3,15]. Within the realm of parametric regression copula-based models, traditional survival distributions—particularly the exponential, Weibull, and Gompertz distributions—remain significant, both theoretically and practically [16]. The primary challenge in parametric copula-based modeling lies in the optimization process, which is complicated by the intricate likelihood structure of semi-competing risks data. This complexity makes the estimation particularly sensitive to the choice of initial values. We implement a two-step estimation approach to address this issue and reduce the dependence on initial value selection. First, we employed the method developed by Shih and Louis [3] to obtain preliminary parameter estimates. These estimates are then used as initial values for the second step maximum likelihood estimation based on the joint likelihood of the two marginal events, thereby improving the stability and reliability of the final parameter estimates. We employ the BOBYQA optimization algorithm and demonstrate the competitive performance of our proposed method in comparison to other existing methods through simulations and real data on HIV/AIDS from the Amsterdam Cohort Study (ACS).

The remainder of this article is organized as follows. In Section 2, we provide a brief description of the motivating data. In Section 3, we introduce the fully parametric copula-based regression models that utilize various copula functions and survival functions. We also explain the main procedure of the state-of-the-art BOBYQA optimization method. Subsequently, we applied our method to the real data on HIV/AIDS from the Amsterdam Cohort Study (ACS) (Section 4) and compared our method with other optimization methods in Section 5. The discussion is given by concluding the article in Section 6.

## 2. Data

The Amsterdam Cohort Study (ACS) on HIV/AIDS among homosexual men is a progressive cohort study that commenced in 1984 to investigate HIV/AIDS. This dataset consists of the time (in years) from HIV infection to AIDS, virus phenotype switching from non-syncytium-inducing to syncytium-inducing (SI switch), and mortality data for 329 men who have sex with men (MSM). Data from the period until combination anti-retroviral therapy are available (1996). The censoring rates for the SI switch event and mortality are approximately 65.7% and 45.9%, respectively. After removing the five partially observed cases, the final dataset comprises 324 samples, and a summary of the variables is listed in Table 1.

This dataset is included in the mstate library and has been analyzed by Geskus et al. [17,18] and Sorrell et al. [19]. However, they only consider the association between events. In this article, we are also interested in the effect of CCR5 and age on the time to SI switch and death events. Due to the sample size limitation, the parametric model may be more suitable, and we specify the marginal distribution for SI switch event and death event.

## 3. Methods

### 3.1. The Copula-Based Model Specifications

In this section, we introduce the model for semi-competing risks events based on the conditional copula function proposed by Patton [20]. For the semi-competing event, we denote $T_{1}$ as the time to a non-terminal event and $T_{2}$ as the time to a terminal event. Moreover, the times $\left(T_{1},T_{2}\right)$ are subject to right censoring $C_{t}$. Then, the time to first event is $X=\min(T_{1},T_{2},C_{t})$, with the censoring indicator $d_{1}=1$ if $T_{1}<\min\left(T_{2},C_{t}\right)$; otherwise, it is $d_{1}=0$. The time to the second event is $Y=\min(T_{2},C_{t})$, with the censoring indicator $d_{2}=1$ if $T_{2}<C_{t}$; otherwise, it is $d_{2}=0$.

We assume the parameters of the copula and marginal survival distribution depend on the covariate vector $Z={\left(Z_{1},\ldots ,Z_{p}\right)}^{⊤}$, where *p* is the dimension of covariates. The marginal survival function is denoted as $S_{T_{1}|Z}\left(t_{1}|z\right)=P\left(T_{1}>t_{1}|z\right)$ for the non-terminal event and $S_{T_{2}|Z}\left(t_{2}|z\right)=P\left(T_{2}>t_{2}|z\right)$ for the terminal event. For the joint survival distribution, we employ a bivariate copula function $C_{\theta }$ with a univariate parameter θ:

(1) $$S\left(t_{1},t_{2}|z\right)=P\left(T_{1}>t_{1},T_{2}>t_{2}|z\right)=C_{\theta }\left(S_{T_{1}|z}\left(t_{1}|z\right),S_{T_{2}|z}\left(t_{2}|z\right)|z\right).$$

Subsequently, the density function for the copula is as follows:

(2) $$c_{\theta }\left(S_{T_{1}|z}\left(t_{1}|z\right),S_{T_{2}|z}\left(t_{2}|z\right)|z\right)=\frac{\partial^{2}C_{\theta }\left(S_{T_{1}|z}\left(t_{1}|z\right),S_{T_{2}|z}\left(t_{2}|z\right)|z\right)}{\partial S_{T_{1}|z}\left(t_{1}|z\right)\partial S_{T_{2}|z}\left(t_{2}|z\right)}$$

and the density corresponding to the survival function can be written as

(3) $$f\left(t_{1},t_{2}|z\right)=c_{\theta }\left(S_{T_{1}|z}\left(t_{1}|z\right),S_{T_{2}|z}\left(t_{2}|z\right)|z\right)f_{T_{1}|z}\left(t_{1}|z\right)f_{T_{2}|z}\left(t_{2}|z\right),$$

where Z, $f_{T_{1}|z}\left(t_{1}|z\right)$ and $f_{T_{2}|z}\left(t_{2}|z\right)$ are the marginal densities of $T_{1}$ and $T_{2}$, respectively.

In this article, we mainly focus on four commonly used univariate-parameter copulas: Clayton, Frank, Gumbel, and normal. To incorporate covariate $Z={\left(Z_{1},\ldots ,Z_{p}\right)}^{⊤}$ effects while maintaining parameter constraints, we employ distinct link functions corresponding to each copula’s parameter space. The four copula families and their respective parameter link functions are formally specified as follows:

(i) *Clayton copula:*
  (4) $$\begin{array}{cc} C_{\theta }\left(u,v\right) & ={\left(u^{-\theta }+v^{-\theta }-1\right)}^{-\frac{1}{\theta }},\;\theta \in \left(0,+\infty \right),\\ \theta & =\exp(b_{0}+b_{1}Z_{1}+\ldots +b_{p}Z_{p}). \end{array}$$
(ii) *Frank copula:*
  (5) $$\begin{array}{cc} C_{\theta }\left(u,v\right) & =-\frac{1}{\theta }\log\left(1-\frac{\left(1-e^{-\theta u}\right)\left(1-e^{-\theta v}\right)}{1-e^{-\theta }}\right),\;\theta \in R\setminus \left\{0\right\},\\ \theta & =b_{0}+b_{1}Z_{1}+...+b_{p}Z_{p}. \end{array}$$
(iii) *Gumbel copula:*
  (6) $$\begin{array}{cc} C_{\theta }\left(u,v\right) & =\exp\left(-{\left[{\left\{-\log\left(u\right)\right\}}^{\theta }+{\left\{-\log\left(v\right)\right\}}^{\theta }\right]}^{1/\theta }\right),\;\theta \in \left[1,+\infty \right),\\ \theta & =\exp(b_{0}+b_{1}Z_{1}+...+b_{p}Z_{p})+1. \end{array}$$
(iv) *Normal copula:*
  (7) $$\begin{array}{cc} C_{\theta }\left(u,v\right) & =\frac{1}{2\pi \sqrt{1-\theta^{2}}}\int_{-\infty }^{\Phi^{-1}\left(u\right)}\int_{-\infty }^{\Phi^{-1}\left(v\right)}exp\left(\frac{-(s^{2}-2\theta st+t^{2})}{2(1-\theta^{2})}\right)dsdt,\;\theta \in \left[-1,1\right],\\ \theta & =\frac{\exp(2\left(b_{0}+b_{1}Z_{1}+...+b_{p}Z_{p}\right))-1}{\exp(2\left(b_{0}+b_{1}Z_{1}+...+b_{p}Z_{p}\right))+1}. \end{array}$$
  where $\Phi^{-1}\left(\cdot \right)$ is the inverse of the cumulative distribution function of the standard univariate Gaussian distribution.

The above three copula functions (i–iii) belong to the Archimedean copula family, and the normal copula (iv) belongs to the elliptical copula family. For more details, we refer the reader to Nelsen [21].

### 3.2. The Parametric Model for Event to Time

For the marginal distribution of non-terminal and terminal events, we consider three popular parametric distributions: exponential, Weibull, and Gompertz. The covariates’ effects on marginal distribution are specified as an exponential linear regression model.

For the exponential marginal distribution, the survival function is given as $S_{T_{i}|Z}\left(t_{i}\right)=\exp\left(-\lambda_{i}t_{i}\right),\;i=1,2,$ for non-terminal and terminal events, respectively. The relationship between parameter $\lambda_{i}$ and covariates $Z={\left(Z_{1},\ldots ,Z_{p}\right)}^{⊤}$ is characterized by the following function:

(8) $$\lambda_{i}=\exp\left(\beta_{i,0}+\beta_{i,1}Z_{1}+\ldots +\beta_{i,p}Z_{p}\right),$$

where $\beta_{i,0},\ldots ,\beta_{i,p}$ are regression coefficients for non-terminal and terminal events, respectively, for i=1,2. We apply the exponential link function to guarantee that $\lambda_{i}$ is positive.

Concerning the Weibull distribution, the survival function can be written as $S_{T_{i}|Z}=\exp\left(-\lambda_{i}t_{i}^{\alpha_{i}}\right)$, where $\lambda_{i}$ and $\alpha_{i}$ are scale and shape parameters, respectively, for i=1,2. We assume the shape parameters $\alpha_{1}$ and $\alpha_{2}$ are constant, and the scale parameters $\lambda_{1}$ and $\lambda_{2}$ are determined by $Z={\left(Z_{1},\ldots ,Z_{p}\right)}^{⊤}$ as

(9) $$\lambda_{i}=\exp\left(\beta_{i,0}+\beta_{i,1}Z_{1}+\ldots +\beta_{i,p}Z_{p}\right),$$

for i=1,2, where $\beta_{i,0},\ldots ,\beta_{i,p}$ are regression coefficients.

As for the Gompertz marginal distribution, the survival function can be written as $S_{T_{i}|Z}\left(t_{i}\right)=\exp\left(-\frac{\lambda_{i}}{\alpha_{i}}\left(\exp\left(\alpha_{i}t_{i}\right)-1\right)\right)$ for i=1,2, where $\lambda_{i}$ is the rate parameter, and $\alpha_{i}$ is the shape parameter. As is the case for the Weibull distribution, let the shape parameter $\alpha_{i}$ be constant and the rate parameter $\lambda_{i}$ depend on $Z={\left(Z_{1},\ldots ,Z_{p}\right)}^{⊤}$ in the following way:

(10) $$\lambda_{i}=\exp\left(\beta_{i,0}+\beta_{i,1}Z_{1}+\ldots +\beta_{i,p}Z_{p}\right),$$

for i=1,2, where $\beta_{i,0},\ldots ,\beta_{i,p}$ are regression coefficients.

### 3.3. Joint Likelihood Function

For the semi-competing bivariate right-censored data, the likelihood based on the copula function $C_{\theta }\left(S_{T_{1}|Z},S_{T_{2}|Z}\right)$ is expressed as

(11) $$\begin{array}{cc} L(\Phi )= & \prod_{i=1}^{n}{\left(c_{\theta }\left(S_{T_{1}|z}\left(t_{i,1}|z_{i}\right),S_{T_{2}|z}\left(t_{i,2}|z_{i}\right)|z_{i}\right)f_{T_{1}|z}\left(t_{i,1}|z_{i}\right)f_{T_{2}|z}\left(t_{i,2}|z_{i}\right)\right)}^{d_{i,1}d_{i,2}}\\ \; & \times {\left(\frac{\partial C_{\theta }\left(S_{T_{1}|z}\left(t_{i,1}|z_{i}\right),S_{T_{2}|z}\left(t_{i,2}|z_{i}\right)|z_{i}\right)}{\partial S_{T_{1}|z}\left(t_{i,1}|z_{i}\right)}f_{T_{1}|z}\left(t_{i,1}|z_{i}\right)\right)}^{d_{i,1}\left(1-d_{i,2}\right)}\\ \; & \times {\left(\frac{\partial C_{\theta }\left(S_{T_{1}|z}\left(t_{i,1}|z_{i}\right),S_{T_{2}|z}\left(t_{i,2}|z_{i}\right)|z_{i}\right)}{\partial S_{T_{2}|z}\left(t_{i,2}|z_{i}\right)}f_{T_{2}|z}\left(t_{i,2}|z_{i}\right)\right)}^{\left(1-d_{i,1}\right)d_{i,2}}\\ \; & \times {\left(C_{\theta }\left(S_{T_{1}|z}\left(t_{i,1}|z_{i}\right),S_{T_{2}|z}\left(t_{i,2}|z_{i}\right)|z_{i}\right)\right)}^{\left(1-d_{i,1}\right)\left(1-d_{i,2}\right)}\\ = & \prod_{i=1}^{n}{\left(c_{\theta }\left(S_{T_{1}|z}\left(t_{i,1}|z_{i}\right),S_{T_{2}|z}\left(t_{i,2}|z_{i}\right)|z_{i}\right)f_{T_{1}|z}\left(t_{i,1}|z_{i}\right)f_{T_{2}|z}\left(t_{i,2}|z_{i}\right)\right)}^{d_{i,1}d_{i,2}}\\ \; & \times {\left(h_{T_{1}|T_{2}}\left(z_{i}\right)\times f_{T_{1}|z}\left(t_{i,1}|z_{i}\right)\right)}^{d_{i,1}\left(1-d_{i,2}\right)}\times {\left(h_{T_{2}|T_{1}}\left(z_{i}\right)\times f_{T_{2}|z}\left(t_{i,2}|z_{i}\right)\right)}^{\left(1-d_{i,1}\right)d_{i,2}}\\ \; & \times {\left(C_{\theta }\left(S_{T_{1}|z}\left(t_{i,1}|z_{i}\right),S_{T_{2}|z}\left(t_{i,2}|z_{i}\right)|z_{i}\right)\right)}^{\left(1-d_{i,1}\right)\left(1-d_{i,2}\right)}, \end{array}$$

where *n* is the sample size, and $c_{\theta }\left(S_{T_{1}|z}\left(t_{i,1}|z_{i}\right),S_{T_{2}|z}\left(t_{i,2}|z_{i}\right)|z_{i}\right)$ is the density function for the copula defined in Equation (2). We assume that the censoring times are independent of the survival times and are not informative.

Here,

$$h_{T_{1}|T_{2}}\left(z_{i}\right)=\left(\frac{\partial C_{\theta }\left(S_{T_{1}|z}\left(t_{i,1}|z_{i}\right),S_{T_{2}|z}\left(t_{i,2}|z_{i}\right)|z_{i}\right)}{\partial S_{T_{1}|z}\left(t_{i,1}|z_{i}\right)}\right)$$

and

$$h_{T_{2}|T_{1}}\left(z_{i}\right)=\left(\frac{\partial C_{\theta }\left(S_{T_{1}|z}\left(t_{i,1}|z_{i}\right),S_{T_{2}|z}\left(t_{i,2}|z_{i}\right)|z_{i}\right)}{\partial S_{T_{2}|z}\left(t_{i,2}|z_{i}\right)}\right)$$

are called h-functions in the literature [22], and the censoring indicator is $d_{i,1}=1$ if $t_{i,1}<\min\left(t_{i,2},C_{t}\right)$; otherwise, it is $d_{i,1}=0$. The censoring indicator is $d_{i,2}=1$ if $t_{i,2}<C_{t}$; otherwise, it is $d_{i,2}=0$. The exact form of the likelihood for the four types of copula functions previously mentioned is given by Wei et al. [23].

### 3.4. Estimation

From Equation (11), we can see that the structure of the log-likelihood function is relatively complex; therefore, it is often a computationally challenging task to estimate the unknown parameters. We define $\Phi ={\left(\alpha^{⊤}=\left(\alpha_{1},\alpha_{2}\right),\beta^{⊤}=\left(\beta_{1}^{⊤},\beta_{2}^{⊤}\right),b^{⊤}\right)}^{⊤}$ as a vector of the parameters, where $\alpha_{j}$ (j=1,2) is the constant parameter of marginal distribution, $\beta_{j}$ (j=1,2) are regression coefficients, and *b* denotes the regression coefficients in the linear predictor of the link function associated with the copula association parameter θ mentioned in Section 3.3. It is known that the terminal event can censor the non-terminal event, so the parameter estimation of the non-terminal event may be biased when using the two-stage method because it treats the censored time as non-informational. Thus, we leverage the popular two-step estimation procedures by Sun et al. [12,24]. The method is shown in Algorithm 1.
**Algorithm 1** Two-step estimation procedure.**Step 1**: Estimate the parameters of marginal distributions and copula function separately.
(1)First obtain the initial values of $\left(\alpha_{j},\beta_{j}\right)$ through grid search method. Then estimate these parameters based on(12)$$\begin{array}{c} \left({\hat{\alpha }}_{j,n}^{\left(1\right)},{\hat{\beta }}_{j,n}^{\left(1\right)}\right)=\underset{\left(\alpha_{j,n},\beta_{j,n}\right)}{\arg\max}\;L_{j,n}\left(\alpha_{j,n},\beta_{j,n}\right), \end{array}$$
where $L_{j,n}$ denotes the likelihood for the marginal survival distribution, j=1,2.(2)Obtain the initial values of $\hat{b}$ through grid search method. Then estimate it by(13)$$\begin{array}{c} {\hat{b}}_{n}^{\left(1\right)}=\underset{b_{n}}{\arg\max}\;L_{n}\left\{{\hat{\alpha }}_{n}^{\left(1\right)}=\left({\hat{\alpha }}_{1,n}^{\left(1\right)},{\hat{\alpha }}_{2,n}^{\left(1\right)}\right),{\hat{\beta }}_{n}^{\left(1\right)}=\left({\hat{\beta }}_{1,n}^{\left(1\right)},{\hat{\beta }}_{2,n}^{\left(1\right)}\right),b_{n}\right\}, \end{array}$$
where ${\hat{\beta }}_{j,n}^{\left(1\right)}$ and ${\hat{\alpha }}_{j,n}^{\left(1\right)}$ are the estimated values by above procedure (1), j=1,2, and $L_{n}$ is the joint likelihood based on the copula function.**Step 2**: Simultaneously maximize the joint likelihood(14)$$\begin{array}{c} {\hat{\Phi }}_{n}=\underset{\Phi }{\arg\max}L_{n}\left\{\alpha_{n}=\left(\alpha_{1,n},\alpha_{2,n}\right),\beta_{n}=\left(\beta_{1,n},\beta_{2,n}\right),b_{n}\right\}, \end{array}$$
with the initial values of ${\hat{\beta }}_{j,n}^{\left(1\right)}$, ${\hat{\alpha }}_{j,n}^{\left(1\right)}$, j=1,2 and ${\hat{b}}_{n}^{\left(1\right)}$ attained from **Step 1**.


In Algorithm 1, the grid search method establishes initial values for parameters $\alpha_{i}$ and $\beta_{j}$ through the following systematic procedure. First, define search ranges for each parameter: $\left[\alpha_{j,lower},\alpha_{j,upper}\right]$ and $\left[\beta_{j,lower},\beta_{j,upper}\right]$. Second, discretize each interval into n equally spaced points: $\alpha_{j,grid}=\left\{\alpha_{j,1},\alpha_{j,2},\ldots ,\alpha_{j,n}\right\}$ and $\beta_{j,grid}=\left\{\beta_{j,1},\beta_{j,2},\ldots ,\beta_{j,n}\right\}$. Third, generate all possible parameter combinations from the Cartesian product of $\alpha_{j,grid}$ and $\beta_{j,grid}$, resulting in $n^{p+1}$ distinct pairs $\left(\alpha_{j,k},\beta_{j,k}\right)$, where *p* denotes the dimension of $\beta_{j}$. Finally, evaluate the marginal survival distribution likelihood $L_{j,k}\left(\alpha_{j,k},\beta_{j,k}\right)$, j=1,2 and $k=1,2,\ldots ,n^{p+1}$ and identify the optimal parameters $\left(\alpha_{j,ini},\beta_{j,ini}\right)=\underset{\left(\alpha_{j,k},\beta_{j,k}\right)}{\arg\max}\left\{L_{j,k}\right\}$. Apply an analogous procedure to determine the initial values for *b*. The parameter estimates derived from Step 1, formally expressed as ${\hat{\Phi }}_{n}^{\left(1\right)}={\left\{{\hat{\alpha }}_{1,n}^{\left(1\right)},{\hat{\alpha }}_{2,n}^{\left(1\right)},{\left({\hat{\beta }}_{1,n}^{\left(1\right)}\right)}^{⊤},{\left({\hat{\beta }}_{2,n}^{\left(1\right)}\right)}^{⊤},{\hat{b}}_{n}^{\left(1\right)}\right\}}^{⊤}$ serve as the initialized parameter vector for the joint optimization process in Step 2.

The accuracy and stability of parameter estimation are critically dependent on appropriate initialization. Direct likelihood maximization in single-step procedures frequently encounters convergence failures when using arbitrary initial values, particularly in complex models. In contrast, the two-stage procedure can be an effective initial method. Given the complexity of the copula itself, the estimation of parameters for the copula and marginal distributions, as outlined in the maximization Equations (12)–(14), is a challenging task. Therefore, we adopt the BOBYQA optimization algorithm instead of the novel limited memory Broyden–Fletcher–Goldfarb–Shanno bounded (L-BFGS-B) method.

The BOBYQA algorithm is a model-based derivative-free trust-region method proposed by Michael J.D. Powell [13]. It is an iterative algorithm used to obtain a minimum of a function $L\left(\Phi \right),\Phi \in R^{p}$, where *p* is the dimension of variable Φ, subject to some box bounds on the variables. It mainly consists of building or updating the interpolation set, constructing or updating the interpolation-based quadratic approximation function, and solving the trust-region subproblem.

At iteration *k*, it employs the interpolation method to construct or update the quadratic model $Q_{k}\left(\Phi \right)$ ($\Phi \in R^{p}$) approximation to the objective function L(Φ) ($\Phi \in R^{p}$) without using its derivatives. For a selected collection of points $\phi_{k}=\left\{\phi_{k,1},\phi_{k,2},\ldots ,\phi_{k,m}\right\}$, $\phi_{k,i}\in R^{p},i=1,2,\ldots ,m$, where *m* is a fixed integer from the interval p+2⩽m⩽(p+1)(p+2)/2, and a local approximation $Q_{k}\left(\phi \right)$ of objective L(ϕ), is constructed as follows:

(15) $$L\left(\phi_{k,i}+s\right)\approx Q_{k}\left(\phi_{k,i}+s\right)=c_{k}+g_{k}^{⊤}s+\frac{1}{2}s^{⊤}H_{k}s,\;s\in R^{p}.$$

and $Q_{k}\left(\phi \right)$ satisfies the interpolation conditions

(16) $$Q_{k}\left(\phi_{k,i}\right)=L\left(\phi_{k,i}\right),\;for\;\phi_{k,i}\in \phi_{k},i=1,2,\ldots ,m.$$

As the number *m* of interpolation points is less than (p+1)(p+2)/2 (the necessary number for a pure interpolation method) and m=2p+1 is recommended by Powell [13,25], the method uses the remaining degree of freedom by solving

(17) $$Q_{k}=\arg\min_{Q}{∥\nabla^{2}Q-\nabla^{2}Q_{k-1}∥}_{F}^{2},$$

where ${∥\cdot ∥}_{F}^{2}$ denotes the square of the Frobenius norm, i.e., ${∥G∥}_{F}^{2}=\Sigma_{i=1}^{m}\Sigma_{j=1}^{n}{\left|G_{ij}\right|}^{2}$ [26]. Further, based on the established quadratic approximation function $Q_{k}\left(x\right)$, it solves its trust-region subproblem. Equally important, the set of *m* interpolation points is also updated during the iterations. The BOBYQA algorithm can be applied to solve functions with large *p* parameters, as mentioned by Ragonneau [27]. When compared with the L-BFGS-B method, the strength of the BOBYQA algorithm is that it can approximate functions whose Hessian matrix is a singular matrix, as also manifested in Section 4. For more information, see [13]. The algorithm is included in the R “optimx” library [28], a replacement and extension of the “optim” function in the “stats” library [29].

As mentioned above, the two-stage procedure refers to an algorithm that optimizes parameters separately, and the two-step procedure denotes an algorithm that optimizes parameters jointly. To evaluate the performance of our proposed TStep-BOBYQA, we compared it with three other methods: TStage-BFGS, TStep-BFGS, and TStage-BOBYQA. TStage-BFGS refers to the optimization of parameters through a two-stage procedure that combines the L-BFGS-B optimization algorithm with initial values obtained via grid search. TStep-BFGS refers to the optimization of parameters using a two-step procedure that integrates the L-BFGS-B optimization algorithm with initial values derived from TStage-BFGS. TStage-BOBYQA refers to the optimization of parameters through a two-stage procedure that combines the BOBYQA optimization algorithm with initial values obtained through grid search. In contrast, TStep-BOBYQA utilizes a two-step procedure coupled with the BOBYQA optimization algorithm, employing initial values sourced from TStage-BOBYQA. These methods are summarized in Table 2.

## 4. Application

In this section, we apply the methods described in Section 3 to the ACS data presented in Section 2. This dataset includes the time to SI switch, time to death from AIDS, the continuous covariate age, and the categorical covariate CCR5. As mentioned in Section 3.2, we utilized three various parametric distributions (i.e., exponential, Weibull, and Gompertz distributions) to model the marginal distribution of survival time. We employed the four different copula functions: Clayton, Frank, Gumbel, and normal, to assess the association between the two survival endpoints. Finally, we selected the best-fitting model based on the Akaike information criterion (AIC). We also assume that the hazard rate and the association between the two events all depend on covariates. We estimate the hazard ratios of the non-terminal and terminal events as well as the regression coefficients of the covariates for the copula function parameters. To eliminate the influence of units, we standardized the times to events and age using a min-max normalization method.

The estimated hazard ratios for each covariate and the regression coefficients of the covariates for the association parameter are reported in Table 3, Table 4, Table 5 and Table 6. The hazard ratios and regression coefficients for the association parameter are similar between the L-BFGS-B methods (TStage-BFGS; TStep-BFGS) and the BOBYQA methods (TStage-BOBYQA; TStep-BOBYQA), with some exceptions. For the Clayton–Gompertz model, parameter estimation can not be obtained using TStep-BFGS (see Table 3). For the Frank–Gompertz model, the variance estimation of regression coefficients of association can not be obtained using TStage-BOBYQA (see Table 4). Additionally, for the Weibull distribution, although the estimated parameters are comparable, the confidence intervals for parameters estimated by the L-BFGS-B methods (TStage-BFGS; TStep-BFGS) are wider than those obtained through the BOBYQA methods (TStage-BOBYQA; TStep-BOBYQA), as illustrated in Table 3, Table 4, Table 5 and Table 6. The methods mentioned above are briefly outlined in Table 2.

From Table 3, Table 4, Table 5 and Table 6, we can see that the two-step methods (TStep-BFGS and TStep-BOBYQA) provide lower AIC values than the two-stage methods (TStage-BFGS and TStage-BOBYQA), with the exception of the copulas combined with the Weibull distribution models. The parameters estimated by the two-stage procedure are identical to those estimated by the two-step procedure, resulting in equivalent AIC values.

### 4.1. Hazard Ratios and Associations

**SI following HIV infection**. Based on the estimation of four different copula functions combined with three different marginal distribution models, no association was found between age and SI switch. Similarly, no association was observed between CCR5 and SI switch, except in two models: the Frank–exponential model (HR:0.705, 95% CI: 0.425 to 0.986, obtained through TStep-BFGS and TStep-BOBYQA; see Table 4) and the normal–Gompertz model (HR: 0.698, 95% CI: 0.402 to 0.995, using TStep-BFGS and TStep-BOBYQA; see Table 6). Thus, the “WM” groups are shown to have a lower risk of SI switch.

**Death following HIV infection**. According to the results of all models, age is not found to be associated with death, as the value of 1 is within their HR confidence intervals. We can see that the CCR5 HR is less than the value of 1, and their HR confidence intervals do not include a value of 1; this means that it is associated with death, and the “WM” groups have a lower risk of death compared to the “WW” groups. These conclusions are consistent for the four different copula models.

**Association between SI and death**. From the results of all models, the association between SI switch and death is stronger for the patients with the CCR5 “WM” genotype than for those with the CCR5 “WW” genotype, except for cases including the Frank–Weibull model, Frank–exponential model, and normal–exponential model. Age does not appear to influence the association for both SI switch and death events, as the value of 0 is within the CI of the age parameter coefficient estimation.

### 4.2. Results for the Preferred Model

For the data from the Amsterdam Cohort Study on HIV infection and AIDS, we employed the AIC to select the optimal model. Our comparative analysis reveals that the two-step estimation method consistently demonstrates superior performance, yielding lower AIC values compared to the two-stage optimization approach in most situations. Among the copula models, the normal copula exhibits the most favorable AIC outcomes, as per Table 3, Table 4, Table 5 and Table 6. Moreover, the Gompertz distribution presents the lowest AIC values within each copula model. These systematic comparisons conclusively identify the normal–Gompertz copula model as the optimal choice for the ACS data.

As demonstrated by the normal–Gompertz survival model in Table 6, individuals with the CCR5 “WM” genotype exhibit significantly reduced risks of both seroconversion illness (SI switch) and mortality compared to the “WW” genotype group. Specifically, the hazard ratios are 0.698 (95% CI: 0.402–0.995) for SI switch and 0.406 (95% CI: 0.235–0.578) for death. This result aligns with the genetic mechanism proposed by Eugen-Olsen et al. [30].

Age demonstrated no statistically significant association with clinical outcomes in the normal–Gompertz survival model. The hazard ratio (HR) for seroconversion illness (SI switch) was 2.065 (95% CI: 0.164–3.967), and the mortality HR reached 3.434 (95% CI: 0.654–6.215), indicating insufficient evidence to guarantee their relationship.

## 5. Simulation Study

In this section, we investigate the performance of our proposed methods through simulations based on different copula functions. On the one hand, we focus on evaluating the estimation accuracy and efficiency of the proposed two-step process and comparing its performances with the two-stage method. On the other hand, we compare the parameter estimation precision achieved by the optimizer BOBYQA (TStage-BOBYQA; TStep-BOBYQA) with that of the popular optimizer L-BFGS-B (TStage-BFGS; TStep-BFGS). The comprehensive simulation results are presented in Table 7, Table 8, Table 9 and Table 10.

### 5.1. Design

The data were generated from various copula models with survival distributions. The main procedure follows the methodology outlined by Wei et al. [23]. We generated 3000 individuals, mimicking the standardized real data analysis from the ACS dataset. The covariate vector is **Z** = ${\left\{Z_{1},Z_{2}\right\}}^{⊤}$, with $Z_{1}$ from Uniform(0, 1) and $Z_{2}$ from Bernoulli(0.3). The parameters of two survival distributions were generated using the exponential regression model, as illustrated in Section 3.2. The times to non-terminal and terminal events were simulated from the exponential, Weibull, and Gompertz survival distributions. The censoring time $C_{t}$ was generated independently from a uniform distribution. The true regression coefficients are specified as in Table 7, Table 8, Table 9 and Table 10. We generated 500 datasets with 3000 individuals.

### 5.2. Performance Measures

To estimate the regression coefficients for the marginal and association parameters, we maximized the likelihood described in Section 3.2 by using the L-BFGS-B optimization algorithm (implemented in the optim function in R) and the BOBYQA algorithm (implemented in the optimx function in R). The performance of our proposed methods was measured using mean squared error (MSE) and coverage probability (CP).

### 5.3. Results

The results are presented in Table 7, Table 8, Table 9 and Table 10. As expected, in the two-stage optimization procedure, the estimation of parameters for the non-terminal regression model is inferior to that of the terminal regression model in terms of MSE and CP, as the non-terminal time to event is censored by the terminal time to event. Furthermore, we can see that the estimation of the parameters for the copula function is suboptimal due to inaccuracies in estimating the parameters for the non-terminal time-to-event regression model. This highlights the limitations of the two-stage optimization method when applied to semi-competing risks data.

When comparing the two-stage and two-step optimization procedures, the coverage probability achieved by the two-step method (TStep-BFGS and TStep-BOBYQA) is closer to the nominal level than that of the two-stage method (TStage-BFGS and TStage-BOBYQA) in most scenarios. Regarding the MSE criteria, the two-step method (TStep-BFGS and TStep-BOBYQA) demonstrates a slight reduction for the exponential distribution with all copula functions when compared to the two-stage method (TStage-BFGS and TStage-BOBYQA) (Table 7). Therefore, the two-step method outperformed the two-stage method.

When comparing the results of the optimization methods, TStage-BFGS yields results similar to those of TStage-BOBYQA with some exceptions. Specifically, for the Clayton–Gompertz and Frank–Gompertz models, TStage-BOBYQA demonstrates a reduction in MSE compared to TStage-BFGS (see Table 7 and Table 8). TStage-BFGS achieves 0.0% coverage probabilities for all coefficients, except for the parameter $\alpha_{NT}$ in the Frank–Weibull model (see Table 8). Furthermore, TStep-BFGS produces results comparable to those of TStep-BOBYQA. It is critical to note that the bounds of parameter values must be specified properly for TStep-BFGS; otherwise, parameter estimation cannot be obtained, rendering it impractical. The BOBYQA optimization method demonstrates greater reliability than the L-BFGS-B optimization method. Consequently, our proposed TStep-BOBYQA optimization method outperforms the other optimization methods in these simulations.

## 6. Discussion

In this article, we present copula-based regression models for semi-competing events with right censoring. We mainly consider four well-known univariate copula functions (i.e., Clayton, Frank, Gumbel, and normal) with three parametric distributions (i.e., exponential, Weibull, and Gompertz) as the marginal distributions. To evaluate the effects of covariates on the time to events, we incorporated these covariates into an interpretable regression model. Due to the complexity of copula-based likelihood functions and the censoring data, estimating the parameters of copula functions and marginal distributions is a great challenge. The initial values of parameters always have a heavy impact on the accuracy of parameter estimation. We employed a two-step procedure method to alleviate the influence and adopted the BOBYQA optimization solver.

In our analysis of real data on HIV/AIDS from the ACS, we employed copula-based models to construct the association between two events. We obtained consistent results, with the exception of the variable CCR5 and its effect on the SI switch event. Specifically, CCR5 shows no relation to the SI switch event in most models, except for two cases: the Frank–exponential model (HR:0.705, 95% CI: 0.425 to 0.986, using TStep-BFGS and TStep-BOBYQA; see Table 4) and the normal–Gompertz model (HR: 0.698, 95% CI: 0.402 to 0.995, using TStep-BFGS and TStep-BOBYQA; see Table 6), which show that CCR5 “WM” is associated with a lower risk of SI switch. Additionally, we conducted several simulations to evaluate the performance of different models based on MSE and CP criteria. In general, in view of MSE criteria, the two-step method performs slightly better than the two-stage method when utilizing both the L-BFGS-B and BOBYQA optimizers. From the aspect of CP, the two-step method consistently outperforms the two-stage method. For the BOBYQA optimizer, it yields nearly the same accurate results as the L-BFGS-B optimizer in certain simulations, including the Gumbel–exponential and Gumbel–Gompertz models in Table 9, as well as the normal–exponential model in Table 10. However, in terms of CP, the BOBYQA optimizer outperforms the L-BFGS-B optimizer in some other simulations. Specifically, the TStage-BFGS method yields a CP further from 95% for the variable coefficients of the terminal regression models in the Clayton–Weibull, Clayton–Gompertz (Table 7), and Frank–Weibull (Table 8) models. Furthermore, in some simulations, careful specification of the bounds is essential for the L-BFGS-B optimizer; otherwise, parameter estimation may not be achievable.

As it is known, one appealing feature of the copula model is that it allows for separate modeling and estimation of the association parameter and marginal distributions, such that the marginal distributions do not depend on the dependency structure. Consequently, the dependency and margins can be estimated separately. However, different distributional choices within the family may induce significantly different dependence structures, as studied by Shih and Louis [3] and Wang and Wells [31]. The methods for selecting appropriate copula models are critical to avoid incorrect association modeling, which can lead to misleading or incorrect conclusions. In addition to the AIC and BIC criteria, Zhu et al. [32] applied the diagnostic method proposed by Chen and Bandeen-Roche [33] to choose the appropriate copula model based on the observed bivariate failure times (T1,T2). Further work could see the use of asymmetric and heavy-tailed distributions for parametric copulas [34]. Therefore, research is warranted to develop a method for copula model selection.

In this article, we mainly consider the BOBYQA derivative-free optimization algorithm. More relevant algorithms surveyed by Rios and Sahinidis [35] need to be investigated, and they may yield better results. In practical applications, the selection of a copula model and the choice of optimizer must be considered for semi-competing risks data. At present, there is no established criterion for selecting an appropriate optimization algorithm for copula models across different semi-competing risks datasets. Optimization methodologies for semi-competing risks models remain a significant and understudied research area, warranting further systematic investigation.

Moreover, there may be issues related to the misspecification of the parametric marginal survival function. To enhance flexibility, semi-parametric or nonparametric models can be considered. Sun and Ding [36] proposed a class of copula-based semiparametric transformation models for bivariate data subject to general interval censoring. For the marginal model, they used the semi-parametric transformation model and approximate the infinite-dimensional nuisance parameters using sieves with Bernstein polynomials. The major challenges in analyzing semi-competing risks data are parameter identifiability, the stability of parameter estimation, and efficiency. Fine [37] used an iteration strategy between estimating parameters of a terminal event and estimating the parameters of copula and non-terminal events. Finally, in the context of real data, there are only two factors: age and CCR5 for AIDS data. Some other unmeasured covariates may influence the time-to-event, and the frailty term can be added for future work, as mentioned by Wei et al. [23].

## Figures and Tables

**Table 1:** Summary of variables in the Amsterdam Cohort Study dataset (N = 324).

Variable	Values
SI Status	
Switch No switch	113 (34.9%) 211 (65.1%)
Mortality	
Death No Death	178 (54.9%) 146 (45.1%)
CCR5 Genotype ^†^	
WW (Wild Wild) WM (Wild Mutant)	259 (79.9%) 65 (20.1%)
Age (years)	
Median (range) Mean (variance)	34.2 (19–58) 34.7 (53.4)

^†^ WW: Wild type allele on both chromosomes; WM: Mutant allele on one chromosome. Data are presented as n (%) unless otherwise specified.

**Table 2:** Summary of proposed and baseline optimization methods.

Method	Initialization	Algorithm	Procedure
**TStage-BFGS**	Grid search	L-BFGS-B	Two-Stage
**TStep-BFGS**	TStage-BFGS result	L-BFGS-B	Two-Step
**TStage-BOBYQA**	Grid search	BOBYQA	Two-Stage
**TStep-BOBYQA**	TStage-BOBYQA result	BOBYQA	Two-Step

TStage: Two-Stage. TStep: Two-Step. L-BFGS-B: Limited-memory Broyden–Fletcher–Goldfarb–Shanno bounded. BOBYQA: Bound Optimization by Quadratic Approximation.

**Table 3:** The parameter estimation results of the Clayton copula model with exponential, Weibull, and Gompertz marginal distributions.

Marginal Distribution	Method/Covariate	Hazard Ratio (95% CI)		Regression Coefficients on Copula Parameter (95% CI)	AIC
		SI Switch	Death		
Exponential distribution	TStage-BFGS				326.599
	Age	1.529 (−0.024, 3.082)	2.040 (0.418, 3.662)	−0.202 (−2.083, 1.680)	
	CCR5 type: WM	0.826 (0.446, 1.205)	0.496 (0.279, 0.714)	1.283 (0.452, 2.114)	
	TStep-BFGS				319.340
	Age	1.635 (0.194, 3.075)	1.775 (0.415, 3.135)	0.082 (−1.639, 1.803)	
	CCR5 type: WM	0.738 (0.440, 1.035)	0.521 (0.310, 0.732)	0.804 (0.039, 1.569)	
	TStage-BOBYQA				326.599
	Age	1.529 (−0.024, 3.082)	2.040 (0.418, 3.662)	−0.202 (−1.200, 0.796)	
	CCR5 type: WM	0.826 (0.446, 1.205)	0.496 (0.279, 0.714)	1.283 (0.978, 1.588)	
	TStep-BOBYQA				319.340
	Age	1.635 (0.194, 3.075)	1.775 (0.415, 3.135)	0.081 (−1.640, 1.802)	
	CCR5 type: WM	0.738 (0.440, 1.035)	0.521 (0.310, 0.732)	0.804 (0.039, 1.569)	
Weibull distribution	TStage-BFGS				256.229
	Age	2.718 (−0.270, 5.706)	3.719 (0.659, 6.778)	0.364 (−1.656, 2.384)	
	CCR5 type: WM	1.000 (0.514, 1.486)	0.404 (0.226, 0.582)	1.397 (0.447, 2.347)	
	TStep-BFGS				256.229
	Age	2.718 (−0.374, 5.810)	3.719 (0.669, 6.768)	0.364 (−1.935, 2.663)	
	CCR5 type: WM	1.000 (0.462, 1.538)	0.404 (0.231, 0.577)	1.397 (0.265, 2.528)	
	TStage-BOBYQA				250.411
	Age	2.718 (0.024, 5.413)	3.719 (0.659, 6.778)	0.235 (−0.558, 1.028)	
	CCR5 type: WM	1.000 (0.561, 1.439)	0.404 (0.226, 0.582)	1.329 (0.975, 1.683)	
	TStep-BOBYQA				250.411
	Age	2.718 (0.041, 5.395)	3.719 (0.723, 6.715)	0.235 (−1.791, 2.261)	
	CCR5 type: WM	1.000 (0.545, 1.455)	0.404 (0.231, 0.577)	1.329 (0.353, 2.305)	
Gompertz distribution	TStage-BFGS				257.348
	Age	1.708 (−0.051, 3.468)	3.708 (0.645, 6.771)	0.484 (−1.384, 2.351)	
	CCR5 type: WM	0.781 (0.420, 1.143)	0.400 (0.224, 0.576)	1.229 (0.363, 2.095)	
	TStep-BFGS				−
	Age	39,610.734 (39,610.734, 39,610.734)	3,269,017.372 (3,269,017.372, 3,269,017.372)	15.000 (15.000, 15.000)	
	CCR5 type: WM	492.465 (492.465, 492.465)	3,269,017.372 (3,269,017.372, 3,269,017.372)	15.000 (15.000, 15.000)	
	TStage-BOBYQA				257.348
	Age	1.708 (−0.051, 3.468)	3.708 (0.645, 6.771)	0.484 (−0.406, 1.373)	
	CCR5 type: WM	0.781 (0.420, 1.143)	0.400 (0.224, 0.576)	1.229 (0.853, 1.606)	
	TStep-BOBYQA				252.517
	Age	2.172 (0.092, 4.251)	3.393 (0.632, 6.155)	0.662 (−1.178, 2.502)	
	CCR5 type: WM	0.707 (0.402, 1.013)	0.400 (0.228, 0.573)	1.025 (0.161, 1.888)	

**Table 4:** The parameter estimation results of the Frank copula model with exponential, Weibull, and Gompertz marginal distributions.

Marginal Distribution	Method/Covariate	Hazard Ratio (95% CI)		Regression Coefficients on Copula Parameter (95% CI)	AIC
		SI Switch	Death		
Exponential distribution	TStage-BFGS				325.685
	Age	1.529 (−0.024, 3.082)	2.040 (0.418, 3.662)	−2.017 (−9.359, 5.324)	
	CCR5 type: WM	0.826 (0.446, 1.205)	0.496 (0.279, 0.714)	5.742 (0.220, 11.264)	
	TStep-BFGS				314.003
	Age	1.422 (0.174, 2.671)	1.611 (0.357, 2.864)	−1.074 (−10.917, 8.769)	
	CCR5 type: WM	0.705 (0.425, 0.986)	0.523 (0.316, 0.729)	4.079 (−1.632, 9.787)	
	TStage-BOBYQA				325.685
	Age	1.529 (−0.024, 3.082)	2.040 (0.418, 3.662)	−2.017 (−2.060, −1.974)	
	CCR5 type: WM	0.826 (0.446, 1.205)	0.496 (0.279, 0.714)	5.742 (5.742, 5.742)	
	TStep-BOBYQA				314.003
	Age	1.423 (0.174, 2.672)	1.612 (0.357, 2.866)	−1.085 (−10.927, 8.757)	
	CCR5 type: WM	0.706 (0.425, 0.986)	0.523 (0.316, 0.729)	4.086 (−1.630, 9.802)	
Weibull distribution	TStage-BFGS				253.969
	Age	2.718 (−0.270, 5.706)	3.719 (0.659, 6.778)	0.795 (−4.983, 6.573)	
	CCR5 type: WM	1.000 (0.514, 1.486)	0.404 (0.226, 0.582)	3.538 (−0.294, 7.371)	
	TStep-BFGS				253.969
	Age	2.718 (−0.433, 5.870)	3.719 (0.529, 6.908)	0.795 (−5.999, 7.589)	
	CCR5 type: WM	1.000 (0.488, 1.512)	0.404 (0.230, 0.578)	3.538 (−0.746, 7.822)	
	TStage-BOBYQA				247.748
	Age	2.718 (0.024, 5.413)	3.719 (0.659, 6.778)	0.873 (0.806, 0.939)	
	CCR5 type: WM	1.000 (0.561, 1.439)	0.404 (0.226, 0.582)	3.433 (3.433, 3.433)	
	TStep-BOBYQA				247.748
	Age	2.718 (0.052, 5.385)	3.719 (0.581, 6.856)	0.873 (−5.731, 7.476)	
	CCR5 type: WM	1.000 (0.564, 1.436)	0.404 (0.230, 0.578)	3.433 (−0.564, 7.431)	
Gompertz distribution	TStage-BFGS				254.023
	Age	1.709 (−0.051, 3.468)	3.708(0.645, 6.771)	0.979(−5.248, 7.205)	
	CCR5 type: WW	0.781 (0.420, 1.143)	0.400 (0.224, 0.576)	3.011 (−0.783, 6.805)	
	TStep-BFGS				249.938
	Age	1.943 (0.121, 3.76)	2.924 (0.474, 5.374)	2.629 (−4.556, 9.814)	
	CCR5 type: WW	0.722 (0.422, 1.023)	0.405 (0.234, 0.576)	2.679 (−1.160, 6.519)	
	TStage-BOBYQA				254.024
	Age	1.708 (−0.051, 3.468)	3.708 (0.645, 6.771)	0.978 (0.851, 1.105)	
	CCR5 type: WW	0.781 (0.420, 1.143)	0.400 (0.224, 0.576)	3.011 (3.011, 3.011)	
	TStep-BOBYQA				249.938
	Age	1.943 (0.122, 3.765)	2.922 (0.474, 5.370)	2.653 (−4.532, 9.839)	
	CCR5 type: WW	0.722 (0.421, 1.023)	0.405 (0.234, 0.576)	2.688 (−1.155, 6.531)	

**Table 5:** The parameter estimation results of the Gumbel copula model with exponential, Weibull, and Gompertz marginal distributions.

Marginal Distribution	Method/Covariate	Hazard Ratio (95% CI)		Regression Coefficients on Copula Parameter (95% CI)	AIC
		SI Switch	Death		
Exponential distribution	TStage-BFGS				330.925
	Age	1.529 (−0.024, 3.082)	2.040 (0.418, 3.662)	0.194 (−2.026, 2.414)	
	CCR5 type: WM	0.826 (0.446, 1.205)	0.496 (0.279, 0.714)	1.180 (0.389, 1.971)	
	TStep-BFGS				323.343
	Age	1.780 (0.263, 3.297)	1.882 (0.404, 3.359)	0.600 (−1.301, 2.501)	
	CCR5 type: WM	0.757 (0.452, 1.063)	0.504 (0.296, 0.713)	0.847 (0.134, 1.560)	
	TStage-BOBYQA				330.925
	Age	1.529 (−0.024, 3.082)	2.040 (0.418, 3.662)	0.194 (−0.405, 0.794)	
	CCR5 type: WM	0.826 (0.446, 1.205)	0.496 (0.279, 0.714)	1.180 (0.313, 2.047)	
	TStep-BOBYQA				323.343
	Age	1.780 (0.263, 3.297)	1.882 (0.404, 3.359)	0.600 (−1.301, 2.502)	
	CCR5 type: WM	0.757 (0.452, 1.063)	0.504 (0.296, 0.713)	0.847 (0.134, 1.560)	
Weibull distribution	TStage-BFGS				251.986
	Age	2.718 (−0.270, 5.706)	3.719 (0.659, 6.778)	−0.112 (−2.204, 1.980)	
	CCR5 type: WM	1.000 (0.514, 1.486)	0.404 (0.226, 0.582)	1.026 (0.195, 1.856)	
	TStep-BFGS				251.986
	Age	2.718 (−0.102, 5.538)	3.719 (0.701, 6.737)	−0.112 (−2.275, 2.051)	
	CCR5 type: WM	1.000 (0.525, 1.475)	0.404 (0.231, 0.576)	1.026 (0.147, 1.905)	
	TStage-BOBYQA				247.238
	Age	2.718 (0.024, 5.413)	3.719 (0.659, 6.778)	−0.150 (−0.511, 0.212)	
	CCR5 type: WM	1.000 (0.561, 1.439)	0.404 (0.226, 0.582)	0.846 (0.479, 1.213)	
	TStep-BOBYQA				247.238
	Age	2.718 (0.177, 5.259)	3.719 (0.718, 6.720)	−0.150 (−2.169, 1.870)	
	CCR5 type: WM	1.000 (0.571, 1.429)	0.404 (0.230, 0.578)	0.846 (0.015, 1.676)	
Gompertz distribution	TStage-BFGS				254.501
	Age	1.709 (−0.051, 3.468)	3.708 (0.645, 6.771)	0.258 (−1.569, 2.086)	
	CCR5 type: WW	0.781(0.420, 1.143)	0.400(0.224, 0.576)	0.804 (0.065, 1.542)	
	TStep-BFGS				252.165
	Age	2.075 (0.187, 3.964)	3.461 (0.627, 6.294)	0.490 (−1.294, 2.274)	
	CCR5 type: WW	0.715 (0.411, 1.019)	0.408 (0.235, 0.581)	0.670 (−0.059, 1.399)	
	TStage-BOBYQA				254.502
	Age3	1.708 (−0.051, 3.468)	3.708(0.645, 6.771)	0.258 (−0.167, 0.684)	
	CCR5 type: WW	0.781 (0.420, 1.143)	0.400 (0.224, 0.576)	0.804 (0.438, 1.169)	
	TStep-BOBYQA				252.165
	Age	2.074 (0.186, 3.962)	3.458 (0.626, 6.290)	0.487 (−1.297, 2.272)	
	CCR5 type: WW	0.715 (0.411, 1.019)	0.408 (0.235, 0.581)	0.670 (−0.059, 1.399)	

**Table 6:** The parameter estimation results of the normal copula model with exponential, Weibull, and Gompertz marginal distributions.

Marginal Distribution	Method/Covariate	Hazard Ratio (95% CI)		Regression Coefficients on Copula Parameter (95% CI)	AIC
		SI Switch	Death		
Exponential distribution	TStage-BFGS				323.288
	Age	1.529 (−0.024, 3.082)	2.040 (0.418, 3.662)	0.143 (−0.859, 1.145)	
	CCR5 type: WM	0.826 (0.446, 1.205)	0.496 (0.279, 0.714)	0.644 (0.222, 1.066)	
	TStep-BFGS				313.698
	Age	1.668 (0.222, 3.114)	1.822 (0.409, 3.235)	0.165 (−0.882, 1.213)	
	CCR5 type: WM	0.735 (0.439, 1.030)	0.506 (0.299, 0.714)	0.426 (0.000, 0.852)	
	TStage-BOBYQA				323.288
	Age	1.529 (−0.024, 3.082)	2.040 (0.418, 3.662)	0.143 (−0.859, 1.145)	
	CCR5 type: WM	0.826 (0.446, 1.205)	0.496 (0.279, 0.714)	0.644 (0.222, 1.066)	
	TStep-BOBYQA				313.698
	Age	1.668 (0.222, 3.114)	1.822 (0.409, 3.235)	0.165 (−0.882, 1.213)	
	CCR5 type: WM	0.735 (0.439, 1.030)	0.506 (0.299, 0.714)	0.426 (0.000, 0.852)	
Weibull distribution	TStage-BFGS				249.868
	Age	2.718 (−0.270, 5.706)	3.719 (0.659, 6.778)	0.042 (−0.852, 0.936)	
	CCR5 type: WM	1.000 (0.514, 1.486)	0.404 (0.226, 0.582)	0.517 (0.086, 0.947)	
	TStep-BFGS				249.868
	Age	2.718 (−0.234, 5.671)	3.719 (0.666, 6.772)	0.042 (−0.930, 1.014)	
	CCR5 type: WM	1.000 (0.507, 1.493)	0.404 (0.231, 0.577)	0.517 (0.056, 0.977)	
	TStage-BOBYQA				244.226
	Age	2.718 (0.024, 5.413)	3.719 (0.659, 6.778)	0.003 (−0.895, 0.901)	
	CCR5 type: WM	1.000 (0.561, 1.439)	0.404 (0.226, 0.582)	0.447 (0.024, 0.870)	
	TStep-BOBYQA				244.226
	Age	2.718 (0.112, 5.324)	3.719 (0.700, 6.737)	0.003 (−0.919, 0.926)	
	CCR5 type: WM	1.000 (0.562, 1.438)	0.404 (0.228, 0.580)	0.447 (0.003, 0.891)	
Gompertz distribution	TStage-BFGS				249.546
	Age	1.709 (−0.051, 3.468)	3.708 (0.645, 6.771)	0.245 (−0.627, 1.117)	
	CCR5 type: WM	0.781 (0.420, 1.143)	0.400 (0.224, 0.576)	0.481 (0.067, 0.896)	
	TStep-BFGS				245.306
	Age	2.065(0.164, 3.967)	3.434 (0.654, 6.215)	0.283 (−0.612, 1.177)	
	CCR5 type: WM	0.698 (0.402, 0.995)	0.406 (0.235, 0.578)	0.353 (−0.061, 0.767)	
	TStage-BOBYQA				249.546
	Age	1.708 (−0.051, 3.468)	3.708 (0.645, 6.771)	0.245 (−0.627, 1.117)	
	CCR5 type: WM	0.781 (0.420, 1.143)	0.400 (0.224, 0.576)	0.481 (0.067, 0.896)	
	TStep-BOBYQA				245.306
	Age	2.065 (0.164, 3.967)	3.434 (0.654, 6.215)	0.282(−0.612, 1.177)	
	CCR5 type: WM	0.698 (0.402, 0.995)	0.406 (0.235, 0.578)	0.353 (−0.061, 0.766)	

The results are for real data on HIV/AIDS from the Amsterdam Cohort Study. The binary covariate CCR5 has two categories: “WM” and “WW” (reference). The SI switch refers to the virus phenotype switching from non-syncytium-inducing to syncytium-inducing.

**Table 7:** Simulation results for the Clayton copula.

Parameters	True_Value	TStage-BFGS		TStep-BFGS		TStage-BOBYQA		TStep-BOBYQA	
		MSE	CP	MSE	CP	MSE	CP	MSE	CP
Exponential distribution									
${\hat{\beta }}_{NT,0}$	1.360	0.046	0.0	0.002	94.4	0.046	0.0	0.002	94.4
${\hat{\beta }}_{NT,age}$	0.577	0.011	81.6	0.004	93.6	0.011	81.6	0.004	93.6
${\hat{\beta }}_{NT,ccr5}$	0.100	0.002	93.4	0.002	94.0	0.002	93.4	0.002	94.0
${\hat{\beta }}_{T,0}$	0.980	0.002	94.2	0.002	94.8	0.002	94.2	0.002	94.8
${\hat{\beta }}_{T,age}$	0.300	0.005	95.0	0.004	94.2	0.005	95.0	0.004	94.2
${\hat{\beta }}_{T,ccr5}$	0.150	0.001	95.8	0.001	95.8	0.001	95.8	0.001	95.8
${\hat{\beta }}_{C,0}$	1.260	0.070	10.4	0.006	93.4	0.071	0.4	0.006	93.4
${\hat{\beta }}_{C,age}$	−0.037	0.054	57.0	0.015	94.4	0.054	33.6	0.016	94.4
${\hat{\beta }}_{C,ccr5}$	−1.200	0.011	89.8	0.008	95.2	0.011	52.2	0.008	95.2
Time (minutes)		171.6		242.1		208.0		315.7	
Weibull distribution									
${\hat{\alpha }}_{NT}$	2.600	0.002	92.8	0.001	95.6	0.002	92.8	0.001	95.8
${\hat{\beta }}_{NT,0}$	−1.660	0.004	87.0	0.002	94.8	0.004	87.0	0.002	94.6
${\hat{\beta }}_{NT,age}$	−0.077	0.004	96.0	0.003	96.2	0.004	96.0	0.003	96.0
${\hat{\beta }}_{NT,ccr5}$	0.210	0.001	97.2	0.001	97.0	0.001	97.2	0.001	97.0
${\hat{\alpha }}_{T}$	2.990	0.020	5.8	0.002	94.0	0.002	95.4	0.002	93.4
${\hat{\beta }}_{T,0}$	−3.260	0.068	0.0	0.005	94.0	0.005	95.4	0.005	93.0
${\hat{\beta }}_{T,age}$	−0.370	0.018	60.8	0.004	94.4	0.005	94.2	0.004	94.4
${\hat{\beta }}_{T,ccr5}$	0.050	0.003	89.4	0.001	97.4	0.001	97.4	0.001	97.4
${\hat{\beta }}_{C,0}$	1.260	0.013	68.6	0.006	95.2	0.006	55.6	0.006	95.2
${\hat{\beta }}_{C,age}$	−0.037	0.054	63.8	0.019	95.6	0.021	54.2	0.020	95.2
${\hat{\beta }}_{C,ccr5}$	−1.200	0.015	87.4	0.011	94.0	0.011	46.2	0.011	94.0
Time (minutes)		850.2		976.4		876.3		1055.1	
Gompertz distribution									
${\hat{\alpha }}_{NT}$	2.600	0.004	91.6	0.002	95.8	0.004	91.6	0.002	95.8
${\hat{\beta }}_{NT,0}$	−1.660	0.005	90.8	0.003	95.4	0.005	90.8	0.003	95.2
${\hat{\beta }}_{NT,age}$	−0.077	0.004	96.0	0.004	96.0	0.004	96.0	0.004	95.8
${\hat{\beta }}_{NT,ccr5}$	0.210	0.002	95.6	0.001	95.8	0.002	95.6	0.001	96.0
${\hat{\alpha }}_{T}$	2.990	0.023	0.7	0.002	94.8	0.003	94.8	0.002	94.0
${\hat{\beta }}_{T,0}$	−3.260	0.068	0.0	0.006	95.2	0.006	94.6	0.005	94.8
${\hat{\beta }}_{T,age}$	−0.370	0.013	75.6	0.004	96.4	0.005	94.4	0.004	94.4
${\hat{\beta }}_{T,ccr5}$	0.050	0.003	88.2	0.002	94.4	0.002	94.2	0.002	94.6
${\hat{\beta }}_{C,0}$	1.260	0.009	83.6	0.006	96.6	0.006	57.2	0.006	96.6
${\hat{\beta }}_{C,age}$	−0.037	0.044	74.8	0.022	94.4	0.023	51.0	0.022	94.4
${\hat{\beta }}_{C,ccr5}$	−1.200	0.014	90.4	0.012	94.6	0.012	45.4	0.012	94.8
Time (minutes)		664.2		785.2		702.4		859.4	

Data were generated using the Clayton copula exponential, Weiull, and Gompertz models, respectively. We generated 500 datasets, with 3000 individuals in each. MSE: mean squared error. CP: empirical coverage probability for 95% confidence interval. TStage-BFGS: two-stage procedure with L-BFGS-B optimization algorithm. TStep-BFGS: two-step procedure with L-BFGS-B optimization algorithm. TStage-BOBYQA: two-stage procedure with BOBYQA optimization algorithm. TStep-BOBYQA: two-step procedure with BOBYQA optimization algorithm.

**Table 8:** Simulation results for the Frank copula.

Parameters	True_Value	TStage-BFGS		TStep-BFGS		TStage-BOBYQA		TStep-BOBYQA	
		MSE	CP	MSE	CP	MSE	CP	MSE	CP
Exponential distribution									
${\hat{\beta }}_{NT,0}$	1.360	0.003	71.0	0.008	97.0	0.003	71.0	0.008	95.0
${\hat{\beta }}_{NT,age}$	0.577	0.007	96.0	0.007	97.0	0.007	96.0	0.007	95.0
${\hat{\beta }}_{NT,ccr5}$	0.100	0.003	71.6	0.007	94.6	0.003	71.6	0.007	94.4
${\hat{\beta }}_{T,0}$	0.980	0.002	96.8	0.002	96.8	0.002	96.8	0.002	96.8
${\hat{\beta }}_{T,age}$	0.300	0.004	94.6	0.004	94.4	0.004	94.6	0.004	94.4
${\hat{\beta }}_{T,ccr5}$	0.150	0.001	97.0	0.001	97.0	0.001	97.0	0.001	97.0
${\hat{\beta }}_{C,0}$	1.260	0.105	91.8	0.098	93.4	0.105	10.6	0.097	93.6
${\hat{\beta }}_{C,age}$	−0.037	0.257	95.0	0.210	93.8	0.256	14.4	0.210	93.8
${\hat{\beta }}_{C,ccr5}$	−1.200	0.229	94.4	0.077	94.6	0.083	21.8	0.077	94.6
Time (minutes)		425.1		570.5		398.7		500.7	
Weibull distribution									
${\hat{\alpha }}_{NT}$	2.600	0.002	95.4	0.002	95.4	0.002	95.0	0.002	95.8
${\hat{\beta }}_{NT,0}$	−1.660	0.004	0.0	0.003	95.2	0.003	92.0	0.003	95.2
${\hat{\beta }}_{NT,age}$	−0.077	0.005	0.0	0.005	95.0	0.006	93.2	0.005	94.4
${\hat{\beta }}_{NT,ccr5}$	0.210	0.002	0.0	0.002	96.4	0.003	89.4	0.002	94.6
${\hat{\alpha }}_{T}$	2.990	0.003	0.0	0.003	92.8	0.002	95.6	0.002	95.6
${\hat{\beta }}_{T,0}$	−3.260	0.005	0.0	0.005	93.6	0.005	94.6	0.005	95.0
${\hat{\beta }}_{T,age}$	−0.370	0.005	0.0	0.005	93.8	0.005	95.4	0.005	95.0
${\hat{\beta }}_{T,ccr5}$	0.050	0.002	0.0	0.002	96.2	0.002	96.0	0.002	96.4
${\hat{\beta }}_{C,0}$	1.260	0.066	0.0	0.067	95.4	0.083	11.6	0.067	91.6
${\hat{\beta }}_{C,age}$	−0.037	0.151	0.0	0.155	96.0	0.196	18.2	0.156	93.0
${\hat{\beta }}_{C,ccr5}$	−1.200	0.061	0.0	0.063	96.0	0.066	17.4	0.063	93.2
Time (minutes)						1106.7		1292.4	
Gompertz distribution									
${\hat{\alpha }}_{NT}$	2.600	0.000	93.8	0.004	94.2	0.003	93.8	0.004	94.2
${\hat{\beta }}_{NT,0}$	−1.660	0.008	92.8	0.004	95.6	0.005	92.8	0.004	95.2
${\hat{\beta }}_{NT,age}$	−0.077	0.039	93.8	0.005	97.0	0.006	93.8	0.005	96.8
${\hat{\beta }}_{NT,ccr5}$	0.210	0.004	91.2	0.002	96.2	0.002	91.2	0.002	96.0
${\hat{\alpha }}_{T}$	2.990	0.000	95.4	0.003	95.4	0.003	95.4	0.003	95.4
${\hat{\beta }}_{T,0}$	−3.260	0.000	95.0	0.007	95.4	0.007	95.0	0.007	95.4
${\hat{\beta }}_{T,age}$	−0.370	0.013	94.6	0.005	95.0	0.005	94.6	0.005	95.0
${\hat{\beta }}_{T,ccr5}$	0.050	0.004	94.6	0.002	94.2	0.002	94.6	0.002	94.2
${\hat{\beta }}_{C,0}$	1.260	0.235	95.0	0.072	95.2	0.071	12.6	0.072	95.6
${\hat{\beta }}_{C,age}$	−0.037	0.280	94.8	0.198	94.8	0.191	13.4	0.196	94.8
${\hat{\beta }}_{C,ccr5}$	−1.200	0.239	93.4	0.074	93.0	0.072	17.6	0.074	92.8
Time (minutes)		919.6		1090.3		919.6		1090.3	

Data were generated using the Frank copula exponential, Weiull, and Gompertz models, respectively. We generated 500 datasets, with 3000 individuals in each. MSE: mean squared error. CP: empirical coverage probability for 95% confidence interval. TStage-BFGS: two-stage procedure with L-BFGS-B optimization algorithm. TStep-BFGS: two-step procedure with L-BFGS-B optimization algorithm. TStage-BOBYQA: two-stage procedure with BOBYQA optimization algorithm. TStep-BOBYQA: two-step procedure with BOBYQA optimization algorithm.

**Table 9:** Simulation results for the Gumbel copula.

Parameters	True_Value	TStage-BFGS		TStep-BFGS		TStage-BOBYQA		TStep-BOBYQA	
		MSE	CP	MSE	CP	MSE	CP	MSE	CP
Exponential distribution									
${\hat{\beta }}_{NT,0}$	1.360	0.016	24.8	0.002	95.6	0.016	24.8	0.002	95.8
${\hat{\beta }}_{NT,age}$	0.577	0.012	76.2	0.004	95.0	0.012	76.2	0.004	95.2
${\hat{\beta }}_{NT,ccr5}$	0.100	0.010	46.6	0.002	94.4	0.010	46.6	0.001	94.6
${\hat{\beta }}_{T,0}$	0.980	0.002	95.2	0.002	94.4	0.002	95.4	0.002	94.6
${\hat{\beta }}_{T,age}$	0.300	0.005	94.8	0.004	94.0	0.005	95.0	0.004	94.2
${\hat{\beta }}_{T,ccr5}$	0.150	0.001	95.2	0.001	95.4	0.001	95.4	0.001	95.6
${\hat{\beta }}_{C,0}$	1.260	0.009	68.0	0.003	93.0	0.009	68.4	0.003	93.2
${\hat{\beta }}_{C,age}$	−0.037	0.016	78.6	0.007	95.0	0.016	72.6	0.007	95.2
${\hat{\beta }}_{C,ccr5}$	−1.200	0.010	81.0	0.004	95.0	0.010	57.6	0.004	95.2
Time (minutes)		560.4		650.3		590.1		748.0	
Weibull distribution									
${\hat{\alpha }}_{NT}$	2.600	0.001	96.2	0.001	95.4	0.001	95.0	0.001	95.0
${\hat{\beta }}_{NT,0}$	−1.660	0.003	94.0	0.003	93.8	0.003	95.4	0.002	95.0
${\hat{\beta }}_{NT,age}$	−0.077	0.004	94.2	0.004	95.2	0.004	94.8	0.004	95.8
${\hat{\beta }}_{NT,ccr5}$	0.210	0.002	95.4	0.002	96.4	0.002	94.0	0.001	94.4
${\hat{\alpha }}_{T}$	2.990	0.002	94.2	0.002	95.8	0.002	94.2	0.002	94.8
${\hat{\beta }}_{T,0}$	−3.260	0.005	93.8	0.005	94.4	0.005	96.0	0.005	95.8
${\hat{\beta }}_{T,age}$	−0.370	0.004	96.0	0.004	93.8	0.004	95.2	0.004	94.2
${\hat{\beta }}_{T,ccr5}$	0.050	0.002	96.8	0.002	96.8	0.002	95.6	0.001	96.2
${\hat{\beta }}_{C,0}$	1.260	0.003	92.4	0.003	94.2	0.003	89.0	0.003	94.8
${\hat{\beta }}_{C,age}$	−0.037	0.010	94.0	0.010	93.8	0.010	74.6	0.010	94.0
${\hat{\beta }}_{C,ccr5}$	−1.200	0.005	94.2	0.005	94.2	0.005	76.4	0.005	95.4
Time (minutes)		1228.1		1332.6		1248.5		1462.7	
Gompertz distribution									
${\hat{\alpha }}_{NT}$	2.600	0.003	94.4	0.003	95.6	0.003	94.8	0.003	95.0
${\hat{\beta }}_{NT,0}$	−1.660	0.004	96.2	0.003	95.6	0.004	94.8	0.003	95.0
${\hat{\beta }}_{NT,age}$	−0.077	0.004	96.0	0.004	95.6	0.005	95.2	0.004	95.4
${\hat{\beta }}_{NT,ccr5}$	0.210	0.002	92.8	0.001	95.4	0.002	93.6	0.001	93.8
${\hat{\alpha }}_{T}$	2.990	0.003	94.6	0.002	94.2	0.003	94.6	0.002	94.8
${\hat{\beta }}_{T,0}$	−3.260	0.007	94.0	0.005	94.4	0.006	94.6	0.005	94.8
${\hat{\beta }}_{T,age}$	−0.370	0.005	94.4	0.005	96.2	0.006	94.0	0.005	94.2
${\hat{\beta }}_{T,ccr5}$	0.050	0.002	95.4	0.001	95.2	0.002	95.2	0.001	95.4
${\hat{\beta }}_{C,0}$	1.260	0.003	94.0	0.003	94.8	0.003	94.0	0.003	94.2
${\hat{\beta }}_{C,age}$	−0.037	0.009	94.8	0.010	95.0	0.010	95.0	0.010	95.2
${\hat{\beta }}_{C,ccr5}$	−1.200	0.005	95.0	0.005	95.0	0.005	95.2	0.005	95.4
Time (minutes)		1044.5		1147.1		1072.8		1276.7	

Data were generated using the Gumbel copula exponential, Weiull, and Gompertz models, respectively. We generated 500 datasets, with 3000 individuals in each. MSE: mean squared error. CP: empirical coverage probability for 95% confidence interval. TStage-BFGS: two-stage procedure with L-BFGS-B optimization algorithm. TStep-BFGS: two-step procedure with L-BFGS-B optimization algorithm. TStage-BOBYQA: two-stage procedure with BOBYQA optimization algorithm. TStep-BOBYQA: two-step procedure with BOBYQA optimization algorithm.

**Table 10:** Simulation results for the Normal copula.

Parameters	True_Value	TStage-BFGS		TStep-BFGS		TStage-BOBYQA		TStep-BOBYQA	
		MSE	CP	MSE	CP	MSE	CP	MSE	CP
Exponential distribution									
${\hat{\beta }}_{NT,0}$	1.360	0.048	0.0	0.002	94.2	0.050	0.06	0.002	94.2
${\hat{\beta }}_{NT,age}$	0.577	0.010	84.6	0.004	94.6	0.011	84.6	0.004	94.6
${\hat{\beta }}_{NT,ccr5}$	0.100	0.030	0.6	0.002	94.6	0.030	0.6	0.002	94.6
${\hat{\beta }}_{T,0}$	0.980	0.002	97.0	0.001	95.4	0.002	95.0	0.001	95.4
${\hat{\beta }}_{T,age}$	0.300	0.005	94.6	0.004	94.6	0.005	94.6	0.004	94.6
${\hat{\beta }}_{T,ccr5}$	0.150	0.001	96.0	0.001	96.0	0.001	96.0	0.001	96.0
${\hat{\beta }}_{C,0}$	1.260	0.013	29.8	0.002	92.8	0.013	29.8	0.002	92.8
${\hat{\beta }}_{C,age}$	−1.037	0.010	78.8	0.005	92.8	0.010	78.8	0.005	92.8
${\hat{\beta }}_{C,ccr5}$	−1.200	0.006	71.6	0.002	94.4	0.006	71.6	0.002	94.4
Time (minutes)		1637.3		1922.3		1704.4		2109.0	
Weibull distribution									
${\hat{\alpha }}_{NT}$	2.600	0.003	85.8	0.003	96.6	0.002	88.2	0.002	94.4
${\hat{\beta }}_{NT,0}$	1.360	0.012	58.4	0.002	95.8	0.012	57.6	0.002	94.6
${\hat{\beta }}_{NT,age}$	0.577	0.007	90.2	0.003	97.2	0.008	86.2	0.004	94.6
${\hat{\beta }}_{NT,ccr5}$	0.100	0.018	13.2	0.001	96.2	0.019	12.4	0.002	95.2
${\hat{\alpha }}_{T}$	2.990	0.002	93.0	0.002	93.8	0.002	94.6	0.002	93.6
${\hat{\beta }}_{T,0}$	0.980	0.005	95.4	0.004	94.8	0.005	94.0	0.004	94.2
${\hat{\beta }}_{T,age}$	0.300	0.005	94.8	0.004	94.4	0.005	95.4	0.004	94.4
${\hat{\beta }}_{T,ccr5}$	0.150	0.002	96.6	0.001	96.0	0.001	97.0	0.001	96.6
${\hat{\beta }}_{C,0}$	1.260	0.001	93.4	0.001	94.0	0.002	90.4	0.002	92.4
${\hat{\beta }}_{C,age}$	−1.037	0.004	95.8	0.004	95.8	0.005	92.6	0.005	93.0
${\hat{\beta }}_{C,ccr5}$	−1.200	0.002	85.8	0.001	96.0	0.002	86.2	0.002	94.2
Time (minutes)		1656.0		1898.2		1705.1		2134.2	
Gompertz distribution									
${\hat{\alpha }}_{NT}$	2.600	0.007	94.6	0.003	94.2	0.007	84.6	0.003	94.2
${\hat{\beta }}_{NT,0}$	1.360	0.019	55.6	0.004	95.8	0.020	55.6	0.004	95.8
${\hat{\beta }}_{NT,age}$	0.577	0.009	84.2	0.004	95.0	0.009	84.2	0.004	95.0
${\hat{\beta }}_{NT,ccr5}$	0.100	0.021	0.9	0.002	94.6	0.021	0.9	0.002	94.6
${\hat{\alpha }}_{T}$	2.990	0.003	95.4	0.003	95.2	0.003	94.4	0.003	95.4
${\hat{\beta }}_{T,0}$	0.980	0.007	93.8	0.006	94.6	0.007	93.8	0.006	94.6
${\hat{\beta }}_{T,age}$	0.300	0.005	95.6	0.004	94.6	0.005	95.6	0.004	94.6
${\hat{\beta }}_{T,ccr5}$	0.150	0.002	97.0	0.002	97.2	0.002	97.0	0.002	97.2
${\hat{\beta }}_{C,0}$	1.260	0.002	91.4	0.002	93.6	0.002	91.4	0.002	93.6
${\hat{\beta }}_{C,age}$	−1.037	0.005	93.2	0.005	93.2	0.005	93.2	0.005	93.2
${\hat{\beta }}_{C,ccr5}$	−1.200	0.003	95.4	0.002	93.2	0.002	95.4	0.002	93.2
Time (minutes)		1920.6		2520.9		1920.6		2520.9	

Data were generated using the normal copula exponential, Weiull, and Gompertz models, respectively. We generated 500 datasets, with 3000 individuals in each. MSE: mean squared error. CP: empirical coverage probability for 95% confidence interval. TStage-BFGS: two-stage procedure with L-BFGS-B optimization algorithm. TStep-BFGS: two-step procedure with L-BFGS-B optimization algorithm. TStage-BOBYQA: two-stage procedure with BOBYQA optimization algorithm. TStep-BOBYQA: two-step procedure with BOBYQA optimization algorithm.

## Data Availability

Data are contained within the article.
